# Oestrogen receptor-negative/progesterone receptor-positive phenotype of invasive breast carcinoma in Japan: re-evaluated using immunohistochemical staining

**DOI:** 10.1007/s12282-018-0898-9

**Published:** 2018-07-31

**Authors:** Hajime Kuroda, Nozomi Muroi, Mitsuhiro Hayashi, Oi Harada, Kazuei Hoshi, Eisuke Fukuma, Akihito Abe, Keiichi Kubota, Yasuo Imai

**Affiliations:** 1 Department of Diagnostic Pathology, Dokkyo Medical University, 880 Kitakobayashi, Mibu, Tochigi 321-0293 Japan; 2 Department of Surgery I, Dokkyo Medical University, Mibu, Japan; 3 Breast Center, Dokkyo Medical University, Mibu, Japan; 4 Department of Pathology, Kameda Medical Center Hospital, Kamogawa, Japan; 5 Department of Breast Surgery, Kameda Medical Center Hospital, Kamogawa, Japan; 6 Department of Surgery II, Dokkyo Medical University, Mibu, Japan

**Keywords:** Oestrogen receptor, Progesterone receptor, Breast carcinoma, Immunohistochemical staining, Re-evaluation

## Abstract

**Background:**

The existence of progesterone receptor (PgR) expression in oestrogen receptor (ER)-negative breast carcinoma is controversial. Here, we re-evaluated ER-negative/PgR-positive (ER−/PgR+) carcinoma cases by immunohistochemical staining (IHC).

**Materials and methods:**

We selected patients who underwent surgery for primary breast carcinoma from our databases at Dokkyo Medical University Hospital and Kameda General Hospital. Among the 9844 patients, the largest series in Japan, 27 (0.3%) were initially diagnosed as ER−/PgR+ breast carcinomas and we re-evaluated by IHC.

**Results:**

The re-evaluated IHC showed that of the 27 patients with the initial results of ER−/PgR+, 12 were ER+/PgR+, 8 were ER−/PgR−, and 7 were ER−/PgR+. ER was negative in 12 of 27 patients (44.4%), and PgR was positive in 8 of 27 patients (29.6%). In our seven re-evaluated and confirmed as ER−/PgR+ cases, the staining proportions of tumor cells were 0% in ER and 1–69% (average 15.8%) in PgR. The average staining proportion of PgR in the re-evaluated ER−/PgR+ phenotype was lower than the initial diagnosis. Histological grading was as follows: grade I, one case; grade II, two cases; grade III, four cases. There were two lymph-node-positive cases.

**Conclusions:**

The ER−/PgR+ phenotype was confirmed after re-evaluation of ER and PgR assessment by a different pathologist. We recommend that pathologists discuss with clinicians, or re-test and re-evaluate ER/PgR expression, particularly in low-grade carcinoma and with a high staining proportion of PgR in the ER−/PgR+ phenotype.

## Introduction

Steroid hormone receptors were shown to be prognostic and predictive markers for breast carcinoma endocrine therapy [1–11]. It is generally suggested that all pathological diagnosed primary breast carcinomas be examined for oestrogen receptor (ER) and progesterone receptor (PgR) protein expression by immunohistochemical staining (IHC). However, the existence of PgR expression in ER-negative breast carcinoma is controversial [2–8]. PgR is a downstream relative of ER, and is regulated by ER, which binds to the oestrogen-responsive element (ERE) located in the promoter region of the PgR gene [12]. Therefore, researchers suggested that ER−/PgR+ is an erroneous result due to a technical artifact, and others reported that such cases are too rare to consider as a true phenotype for ER and PgR assessments in IHC-based methodology [2–4]. However, it is also reported that downregulation of PgR was not mediated by a reduction in ER levels or ER activity, suggesting that regulation of PgR is independent of ER under some conditions [13]. Thus, other studies suggested that ER−/PgR+ breast carcinomas show definite clinical and biological features [5–8]. Herein, we re-evaluated all the ER−/PgR+ carcinoma cases which we have encountered according to the standard IHC methods. The aim of our study was to determine the existence of the ER−/PgR+ breast carcinoma phenotype and, if found, to elucidate its clinicopathological features and discuss management.

## Methods

### Immunohistochemistry

We selected patients who underwent surgery for primary breast carcinoma following ER/PgR IHC evaluations from our databases at Dokkyo Medical University Hospital and Kameda General Hospital. Surgical and biopsy specimens were fixed in 10% buffered neutral formalin solution for IHC at our hospitals. The immunohistochemical procedures used for the initial staining at Dokkyo Medical University Hospital and Kameda Medical Center Hospital were as follows: in Kameda Medical Center Hospital, the sections were taken to an automated stainer (DAKO, AUTOSTAINER) following the manufacturer’s instructions before 2012. The sections were taken to an automated stainer (VENTANA, BENCHMARK XT) from 2012 to 2017. The evaluated IHC assays were ER (clone 1D5, Dako, 1:50, nuclear), and PgR (clone PgR636, 1:800, nuclear) before 2012, and ER (clone SP1, Ventana, prediluted, nuclear), and PgR (clone 1E2, prediluted, nuclear) from 2012 to 2017. In Dokkyo Medical University Hospital, the evaluated IHC assays were ER (clone 1D5, Dako, 1:100, nuclear), and PgR (clone PgR636, 1:200, nuclear) before 2006. The sections were then taken to an automated stainer (VENTANA, BENCHMARK XT) following the manufacturer’s instructions from 2006 to 2017. The evaluated IHC assays were ER (clone 6F11, Ventana, prediluted, nuclear), and PgR (clone 16, prediluted, nuclear) from 2006 to 2009 and ER (clone SP1, Ventana, prediluted, nuclear), and PgR (clone 1E2, prediluted, nuclear) from 2006 to 2009. Details of the initial cut-off points for ER and PgR are unknown for both hospitals.

**Fig. 1:**
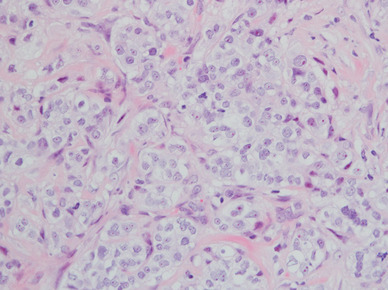
Infiltrating breast carcinoma (HE staining, ×100)

**Fig. 2:**
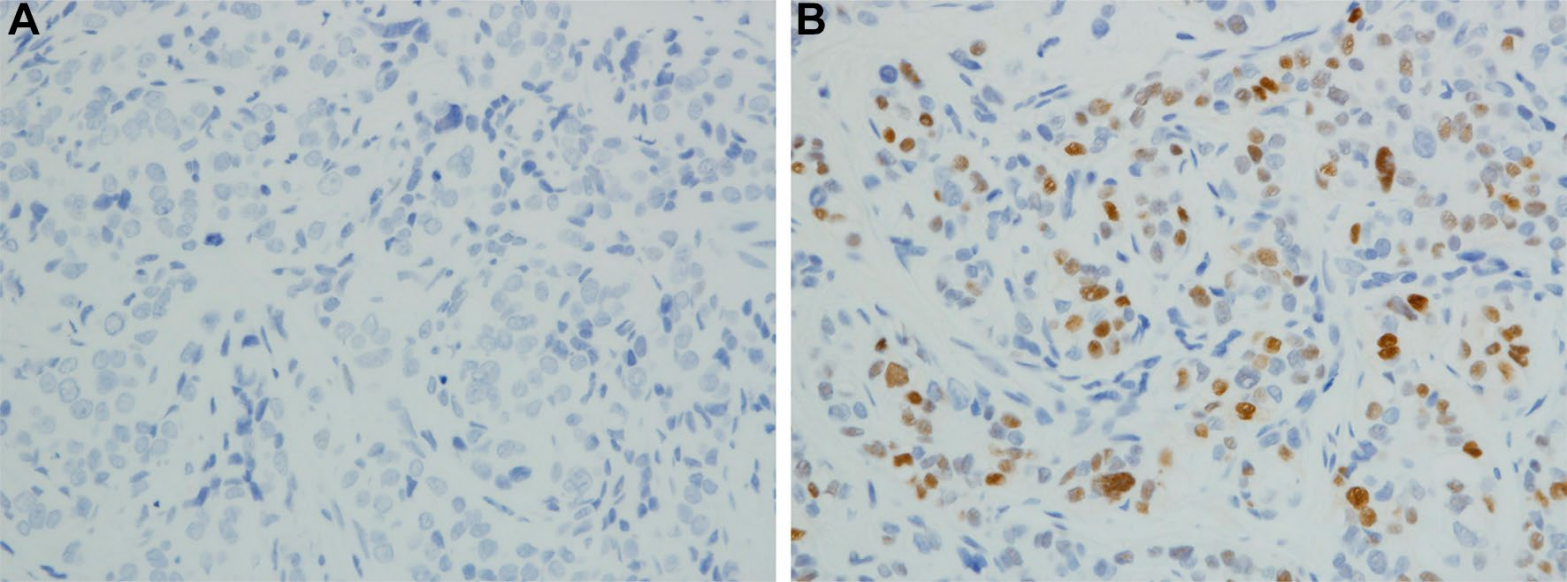
**a** Oestrogen receptor (ER)-negative and **b** progesterone receptor (PgR)-positive breast carcinoma (immunohistochemical staining, ×100)

Among the 9844 patients, 27 (0.3%) were initially diagnosed as ER−/PgR+ breast carcinomas. The clinical history, pathological reports, and haematoxylin and eosin (H&E) slides from all 27 patients were reviewed. ER−/PgR+ carcinomas were re-evaluated by nuclear staining of ER and PgR immunohistochemically at the Department of Diagnostic Pathology of Dokkyo Medical University. In each case, the same block was selected for the IHC re-evaluation. The paraffin-embedded tissue block was recut into 5 µm sections. The re-evaluated IHC assays for ER (clone 6F11, Novocastra, 1:40, nuclear) and PgR (clone 16, Novocastra, 1:100, nuclear) were performed by additional H&E staining. The slides were treated with methanol containing 0.3% hydrogen peroxide to block endogenous peroxidase activity. Antigen retrieval was achieved with microwave treatment for markers. After incubation with the primary antibody, incubation with a secondary biotinylated antibody was performed for 15 min. After washing, the sections were incubated with streptavidin–peroxidase for 20 min. Finally, the enzyme was visualized after a 5-min incubation with diaminobenzidine. Counterstaining was performed with haematoxylin. The methodology was the same in ER and PgR. The re-evaluated ER, PgR, and H&E slides (Fig. 1) were reviewed by two pathologists independently. To increase reproducibility, ER and PgR positivity was defined as 1% nuclear staining of tumor cells (Fig. 2a, b) [14, 15]. As HER2-positive breast carcinomas appear as a biologically distinct phenotype, we excluded HER2-positive cases from this study. The Chi-square test was used to assess associations among the variables, and the Mann–Whitney test was used to compare the means of clinicopathological data. The associations of ER and PgR expression were analysed using Chi-square test and Fisher’s exact test to assess whether there were significant differences in expression. Differences were considered significant when *P* was less than 0.05.

## Results

The results of clinicopathological and re-evaluated IHC for ER and PgR are summarized in Table 1. The clinicopathological findings of the 27 patients with the initial results of ER−/PgR+ were as follows: the patients’ ages ranged from 39 to 73 years (mean age 55.8 years), and tumor size ranged from 0.1 to 11.7 cm (mean size 1.8 cm). Histologic types were 25 invasive carcinomas of no special type (NST), and 2 other cases. Histological grading was as follows: grade I, 12 cases; grade II, 6 cases; grade III, 9 cases. There were 7 lymph-node-positive cases. Tumor staging was as follows: stage I, 16 cases; stage II, 8 cases; stage III, 3 cases. The carcinomas recurred in four cases within the follow-up period of 60 months. These included one case of local recurrence, and three cases of distant metastasis (lymph node, bone, lung, and liver). Two patients died of disease. The re-evaluated IHC showed that of the 27 patients with the initial results of ER−/PgR+, 12 were ER+/PgR+, 8 were ER−/PgR−, and 7 were ER−/PgR+. ER was negative in 12 of 27 patients (44.4%), and PgR was positive in 8 of 27 patients (29.6%). In our seven re-evaluated and confirmed as ER−/PgR+ cases, the staining proportions of tumor cells were 0% in ER and 1–69% (average 15.8%) in PgR. The average staining proportion of PgR in re-evaluated ER−/PgR+ phenotype was lower than the initial diagnosis.

**Table 1:** Clinicopathologic findings of 27 patients with an initial ER−/PgR+ diagnosis

	A. Initial diagnosis		B. Different from initial diagnosis				C. Confirmed initial diagnosis		B vs C
	ER−/PgR+ (27 cases)		ER+/PgR+ (12 cases)		ER−/PgR− (8 cases)		ER−/PgR+ (7 cases)		*P* value
Mean age	55.8 (39–79)		47.8 (39–64)		65.1 (49–79)		56.9 (42–72)		0.9679
Mean tumor size (cm)	1.8 (0.1–11.7)		1.0 (0.1–2.3)		2.9 (0.3–11.7)		1.7 (0.5–3.5)		0.9993
Histology	NST 25 (92.6%)		NST 12 (100.0%)		NST 6 (75.0%)		NST 7 (100.0%)		0.3846
	Others 2 (7.4%)		Others 0 (0%)		Others 2(25.0%)		Others 0 (0%)		
Histological grading									
I	12 (44.4%)		9 (75.0%)		2 (25.0%)		1 (14.3%)		0.2800
II	6 (22.3%)		2 (16.7%)		2 (25.0%)		2 (28.6%)		
III	9 (33.3%)		1 (8.3%)		4 (50.0%)		4 (57.1%)		
Status of nodal metastasis	7 of 27 (25.9%)		4 of 12 (33.3%)		1 of 8 (12.5%)		2 of 7 (28.6%)		0.8528
Stage									
I	16 (59.3%)		8 (66.6%)		5 (62.5%)		3 (42.9%)		0.2500
II	8 (29.6%)		2 (16.7%)		2 (25.0%)		4 (57.1%)		
III	3 (11.1%)		2 (16.7%)		1 (12.5%)		0 (0%)		
IV	0 (0%)		0 (0%)		0 (0%)		0 (0%)		
5 year follow up data available cases	26 of 27		11 of 12		8 of 8		7 of 7		
Local recurrence	1 (3.7%)		0 (0%)		0 (0%)		1 (14.3%)		0.0929
Distant metastasis	3 (11.1%)		2 (16.6%)		0 (0%)		1 (14.3%)		0.7901
Died of disease	2 (7.4%)		1 (8.3%)		0 (0%)		1 (14.3%)		0.4438
Re-evaluated proportion of positive cells	ER	PgR	ER	PgR	ER	PgR	ER	PgR	
	19.3%	28.8%	52.4%	66.9%	0%	0%	0	15.8%	

*ER* oestrogen receptor, *PgR* progesterone receptor, *NST* invasive carcinoma of no special type

The clinicopathological findings of the seven patients with re-evaluated results of the ER−/PgR+ phenotype were as follows. The patients’ ages ranged from 42 to 72 years (mean age 56.9 years). Tumor size ranged from 0.5 to 3.5 cm (mean size 1.7 cm). Histological grading was as follows: grade I, one case; grade II, two cases; grade III, four cases. There were two lymph-node-positive cases. Tumor staging was as follows: stage I, three cases; stage II, four cases. Carcinomas recurred in two cases within the follow-up period of 60 months, including one case of local recurrence and one case of distant metastasis (bone). One patient died of disease.

## Discussion

There are reports, suggesting that the ER−/PgR+ phenotype does not exist and may be a technical artifact [2–4]. Nadji et al. evaluated a large series of 5993 breast carcinomas for ER expression by IHC analysis and found that the ER−/PgR+ phenotype did not exist [2]. Furthermore, several studies suggested that there was no ER−/PgR+ phenotype using re-evaluated IHC (Table 2). De Maeyer et al. found that none of their 32 cases initially considered to be the ER−/PgR+ phenotype were found to be so following a re-evaluated IHC test [3]. Maleki et al. also found that none of their 43 cases initially diagnosed as ER−/PgR+ phenotype showed the same IHC phenotype upon re-evaluation [4]. Ahmed et al. investigated a large number of ER−/PgR+ phenotype samples by re-evaluating IHC using a tissue microarray (TMA) [16]. In contrast to the previous studies, 92 of 267 cases were confirmed as ER−/PgR+. One limitation of this study is that they used the TMA method. The tumor heterogeneity, the data from TMA analysis may not be identical to the results from whole sections.

**Table 2:** Articles on ER−/PgR+ breast cancer

Article	Year	No.	ER−/PgR+ cases in medical record	Re-evaluated ER−/PgR+ cases	Method
De Maeyer et al. [3]	2007	2013	32	0 (0%)	IHC
Maleki et al. [4]	2012	2432	43	0 (0%)	IHC
Ahmed et al. [14]	2016	8315	267	92 (34.6%)	IHC (TMA)
Current	2017	9844	27	7 (25.9%)	IHC

*ER* oestrogen receptor, *PgR* progesterone receptor, *IHC* immunohistochemical staining, *TMA* tissue microarray

These differences in the previous articles may be due to the diagnostic criteria or the methods used for preparing the IHC. The recommended cut-off points of IHC have changed over several decades. Ogawa et al. suggested a 10% staining proportion may be an acceptable cut-off point for both ER and PR status by IHC [17]. However, among the cases initially scored as negative for ER and PgR, the proportion of positive cells in our re-evaluation was 0 or nearly 0%. Furthermore, an important issue is selection of the most reliable antibody. In some of our initial cases, monoclonal anti-ER 1D5 was used for immunohistochemical assessment of ER. Bogina et al. demonstrated the higher sensitivity of anti-ER SP1 and 6F11 clones compared with the 1D5 clone [18]. This may be the cause of the discrepancy in the ER results.

However, we must start from the reality that the methods of preparing IHC specimens differ in each institution and cannot be unified. Thus, it is wiser to accept the existence of the ER−/PgR+ phenotype, and then discuss management. Furthermore, in our study, the ER−/PgR+ phenotype was confirmed after the re-evaluation of ER and PgR by different pathologists. Therefore, regardless of the diagnostic criteria or the process for preparing specimens, we may find ER−/PgR+ breast cancer cases in our routine practice. Moreover, there were several reports showing the gene-expression profile of ER−/PgR+ breast carcinomas [19, 20]. Itoh et al. reported that 25% of cases showed the same IHC phenotype after re-evaluation by gene-expression profiling [19]. Taken together, these results provide further evidence that some ER−/PgR+ cases do not reflect a technical artifact, but are a distinct group of breast carcinomas.

As the ER−/PgR+ phenotype is extremely rare and mostly non-reproducible, the majority of cases classified as ER−/PgR+ may appear as different classifications [16]. The difference between the results of the initial and the re-evaluated IHC raises a concern about possible errors in pathological diagnosis. In our study, most of the initially evaluated ER−/PgR+ cases were ER+/PgR+ low-grade carcinomas after re-evaluation by IHC. Furthermore, in our results, PgR was negative in 8 out of 27 patients (29.6%), which differed from the initial diagnosis. Moreover, in the confirmed ER−/PgR+ cases, the staining proportion of PgR+ was low. Therefore, we recommend that pathologists discuss with clinicians, or re-test and re-evaluate ER/PgR expression, particularly under the following conditions. (1) Histological grade is low. (2) PgR-positive proportion is high.

Ng et al. reported that the ER−/PgR+ phenotype had no distinct histopathological characteristics compared to the ER+/PgR+ and ER−/PgR− phenotypes and no prognostic impact [7]. In contrast, Rakha et al. reported poorer survival of ER−/PgR+ cases compared with ER+/PgR+ cases [5]. Furthermore, Purdie et al. reported that PgR expression is an independent prognostic variable more powerful than ER [21]. PgR expression in breast carcinoma may potentially define a distinct subgroup with paired function in the ER pathway that will probably benefit from endocrine therapy [2–4]. However, we could not draw any conclusions regarding histological characteristics or prognostic impact from the current study due to the small number of cases. It is natural that there is a difference in prognosis outcomes among the previous papers. This is because not only is the ER−/PgR+ phenotype extremely rare, but also there are no unified diagnostic criteria and specimen preparation methods as described above. The ER−/PgR+ phenotype can result in difficulties in examining biological behavior and deciding on an appropriate treatment strategy.

In conclusion, among the 9844 patients, the largest series in Japan, 27 (0.3%) were initially diagnosed as ER−/PgR+ breast carcinomas, and the ER−/PgR+ phenotype was still present after the re-evaluation of ER and PgR using whole sections recut from the same tissue block. However, 20 of 27 patients with the initial results of ER−/PgR+ differed from the initial diagnosis. We recommend that pathologists discuss with clinicians, or re-test and re-evaluate the ER/PgR expression, particularly in low-grade carcinoma and with a high staining proportion of PgR in the ER−/PgR+ phenotype.
